# Autosomal dominant polycystic kidney

**DOI:** 10.11604/pamj.2022.42.270.36599

**Published:** 2022-08-11

**Authors:** Rugaved Raghavendra Gudadhe, Gaurav Rajendra Sawarkar

**Affiliations:** 1 Department of Rachana Sharir, Mahatma Gandhi Ayurved College Hospital and Research Centre, Datta Meghe Institute of Medical Sciences (Deemed to be University) Salod (H), Wardha, Maharashtra, India

**Keywords:** Polycystic kidney, dialysis, renal failure, nephrectomy

## Image in medicine

Polycystic kidney disease is a hereditary illness that causes cystic growth of the kidneys, resulting in increasing kidney enlargement and renal insufficiency, as well as a variety of extrarenal symptoms. The illness has autosomal dominant and recessive inheritance patterns, characterized by gradual but increasing enlargement of the kidneys, with renal failure developing by the fifth to sixth decade of life. A 46-year-old male patient with abdominal discomfort in the flanks for the past 6-7 years, an old medical record suggesting chronic kidney disease, a recent ultrasonography (USG) abdomen, and pelvis confirming polycystic kidney disease, Sr. creatinine has risen to 08 mg/dl. A kidney transplant was advised for the patient. His nephrectomy was recently completed, and he is now on dialysis and supportive drugs, with an emphasis on an early transplant.

**Figure 1:**
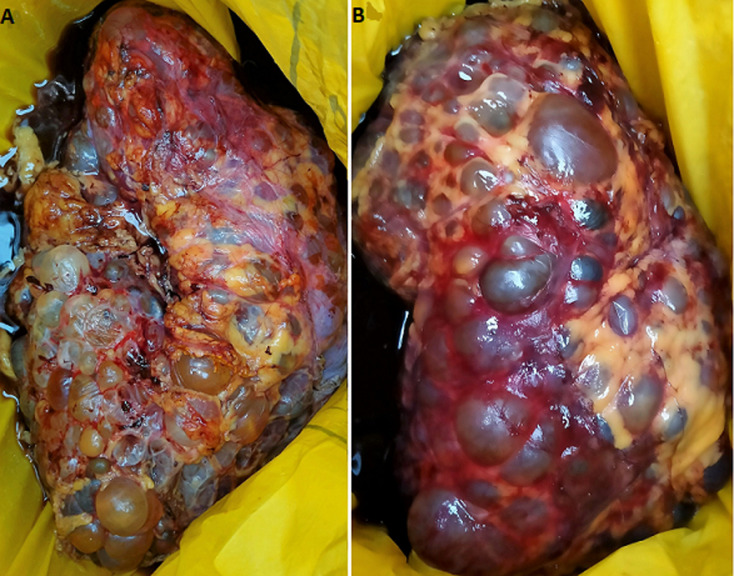
A) right kidney with polycystic kidney disease; B) left kidney with polycystic kidney disease

